# The influence of institutional discourses on the work of informal carers: an institutional ethnography from the perspective of informal carers

**DOI:** 10.1186/s12913-017-2591-7

**Published:** 2017-09-07

**Authors:** Guro Wisth Øydgard

**Affiliations:** Nord University, Box 1490, 8049 Bodø, Norway

**Keywords:** Informal carers, Dementia, Institutional discourse, Allocation of healthcare, Institutional ethnography

## Abstract

**Background:**

The growing numbers of seniors worldwide and the need for support and services that follow from a higher standard of living have led to an increased focus on scarce benefits and limited human resources. At the same time, many western countries have had to make welfare cuts to balance budgets. This has brought the contributions of informal carers to the fore. Thus far, the focus has generally been on the need for the informal carers to receive information and support; to *enable* them to contribute.

**Methods:**

The study is designed as an institutional ethnography. The article describes the social processes of informal caregiving and how it interacts with formal caregiving, from the perspective of informal carers. The research question for the study is *How do institutional discourses on the work of informal carers influence informal carework?* Data for the article comes from qualitative semi-structured interviews with 26 informal carers caring for persons with dementia in Norway, and with 7 administrators working in the allocation divisions of five different municipalities.

**Results:**

The results demonstrate how three institutional discourses of informal carers’ work influence the allocation divisions’ practices and the work of informal carers in caring for their next of kin. The three discourses are categorised as *moral and family obligation, shared care* and *task specificity*. The informal carers want to contribute, as they feel a family and moral obligation to their next of kin. In the interaction with the allocation division, they find that the expectation that they will share in the carework and perform specific tasks forces them to perform care within a framework set by the public services.

**Conclusions:**

The findings suggest that further research should challenge how services are distributed and allocated rather than focus on how to enable informal carers to fulfil their role better. Because of their moral and family obligation, the informal carers do not have to be forced to perform certain tasks or parts of the shared care. To maintain the informal carers’ carework and to fully utilise their contributions, public services would benefit from collaborating with the informal carers to fulfil the total care need of the person with dementia.

## Background

It is well known that caring for family members with dementia is challenging for informal carers. At the same time, there is broad agreement that informal carers are essential to managing the care needs for persons with dementia [1–8]. Until now, much of the focus in the field has been on training and information to enable informal carers to handle their role and challenges as informal carers [1, 3, 9]. However, this study finds that training and information are insufficient to solving the challenges informal carers face. This article explores the work informal carers do in caring for their next of kin and how their work is shaped by institutional discourses on the work of informal carers. The findings show that three institutional discourses on the work of informal carers influence the informal carers’ actions, and that the problem for the informal carers is not caring for their next of kin, but being forced to perform the care within a framework set by the public services.

### A renewed focus on informal carers’ contribution and care

Dementia is a global public health challenge, and caregiving for persons with dementia is of current interest. The challenges in the field revolve around how to manage people with dementia’s increased need for assistance and the cost of this assistance [7, 8, 10]. The growing numbers of seniors worldwide and the need for support and services that follow from a higher standard of living have led to an increased focus on scarce benefits and limited human resources [2, 5, 11]. At the same time, many western countries have had to make welfare cuts to balance budgets. In turn, this has made the importance of informal carers as resources a current issue [7, 8, 10].

In Norway and the other Nordic countries, the work of informal carers is subject to much current interest, in particular because it challenges the tradition of a comprehensive welfare state. The Norwegian welfare system is based on universalism, but like in the other Nordic countries, this universalism is challenged by a new era of growing needs for care. The development of the Norwegian welfare state has been closely connected to the primacy of work. It has been a primary goal that everyone – men and women – should contribute to the community through paid work. Therefore, it is the welfare state that is supposed to be the main provider of care and assistance to those in need, not family members [12]. Informal carers do not have any legal obligation to care for next of kin above the age of 18. The public welfare system bears full responsibility for caring for adults and elderly in need [3].

Nevertheless, most informal carers feel *a moral or family obligation* to care for their next of kin, and informal carers contribute a considerable part of overall care for persons with dementia [3, 5]. This sense of obligation has historical roots, from the time before the massive expansion of the welfare state that took place in Norway in the 1960s and 1970s. Yet despite the massive expansion of public services, research shows that family care has continued. [3] In fact, according to the Ministry of Health and Care Services [5], informal carers contribute approximately the same amount of care as formal care services. Nevertheless, as the welfare state has expanded, a complementarity has developed between formal and informal care. According to Kaasa [3], informal carers help out with the more practical tasks, while the public services provide medical care and nursing. Kröger [2] calls such a division of tasks between formal and informal care a *task specificity model*. This kind of *shared care* seems to be the most common model in both Norway and the other Nordic countries.

Informal carers are also considered important contributors who notify the formal care system when help is needed. This is crucial, as the formal care system relies on applications for help [3, 6]. The latest government documents about care for the elderly in Norway [3–5] do not problematize the shared care model or the role of informal carers in this. Nevertheless, the role of informal carers in caring for the elderly is referred to as crucially important [5]. Informal carers are referred to as resources and collaborators for the formal care services [3]. According to the Ministry of Health and Care Services [5], one of the planned solutions for the increasing numbers of elderly and people living with illness is to share the caregiving between formal and informal carers, or as the white paper says: “*the totality of society’s care resources*.”

### To be an informal carer to a person with dementia

Care is a term with many different meanings. Etters, Goodall [8] define caregiving as “activities and experiences involved in providing help and assistance to relatives who are unable to provide for themselves”. This does not include any psychological distress that may arise from caregiving and having a sick family member. The term “caregiver burden”, on the other hand, includes this psychological distress: “a multidimensional response to physical, psychological, emotional, social and financial stressors associated with the caregiving experience” [8]. Informal carers to persons with dementia are mainly spouses or children. Nevertheless, the term “informal carer” does not only describe relatives, but also other close relatives or friends providing care [7, 8].

Dementia is a progressive illness that leads to an increasing need for help. The Berger [13] scale illustrates how the functional level declines as the illness progresses. Thus, as the illness progresses, the need for help gradually expands. Caring for family members with severe diseases such as dementia can lead to increased health risks, and international research about informal caregivers has traditionally focused on improving individual factors to lighten the caregivers’ burden [7, 8]. Norwegian government documents [3, 5, 6] mention the risk of health injury, but claim that given the extensive formal care system, the risk is limited [5].

Previous research on access to formal services shows that a main challenge for persons with dementia and their informal carers is to navigate the system of formal and informal care. Several studies suggest that there is an unfulfilled need for information among persons with dementia and their informal carers [14–16], as well as among healthcare workers [17–19]. As a result, training and information are provided to informal carers in many European countries [1]. In Norway, the training of and information for informal carers has been a priority area. For informal carers to persons with dementia, this is organised as a special training programme tailored for this specific group of informal carers. The goal is to *enable* informal carers to help care for their life partner or parents longer and without injury to their own health. The education programme has the same design all over Norway, and includes six classes. The content of each class is partly up to the course leaders, but some topics are mandatory. One of the mandatory topics is information about different kinds of health and care services [9]. Yet the training programme provides selective knowledge and presumes a particular way of providing informal care and the allocation of formal services in turn presumes this particular way of providing informal care. The consequence of both the training programme and the allocation practice is therefore to emphasise the informal carer’s role as a provider of care, but this is done by ensuring that informal carers adapt the care they provide to the structures and tasks that have been defined in advance by the formal services. The research described in this article, shows that this particular way of providing informal care is linked to institutional discourses on informal carers’ work. This article contributes to the field by exploring how three institutional discourses about the work of informal carers impact the actions of informal carers.

## Methods

The approach to data collection is inspired by Dorothy Smith [20] and institutional ethnography. This is because institutional ethnography is well suited for investigations of how informal careworkers do carework. Institutional ethnography has a bottom-up perspective, and starts with the people who have first-hand knowledge, who are referred to as “the knowledgeable” [20]. Institutional ethnography makes visible how individual lives are connected with the rest of the social processes. In this article, the social processes of informal caregiving, and their interaction with the allocation of formal caregiving, are described from the perspective of the informal caregivers. The research question for the study is *How do institutional discourses on the work of informal carers influence informal carework?*

**Fig. 1:**
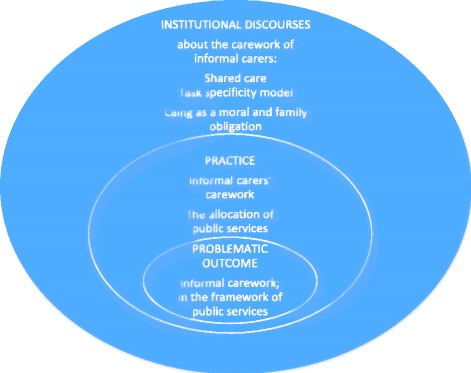
The influence of institutional discourses on the work of informal carers: The figure shows how I progressed from the problematic, seen from the perspective of informal carers, to practice, which allowed me to see how institutional discourses about the carework of informal carers influence both administrators’ allocation practices and the carework provided by informal carers

As dictated by the methods of institutional ethnography, the study started with those who are knowledgeable about the topics that are found to be relevant to investigate [20]. In the present case, this means informal carers who care for persons with dementia. The informal caregivers could share their first-hand knowledge about their role and efforts related to care and services for the person with dementia. The informal carers’ knowledge is what Smith [20] calls “work knowledge”. The mapping of work knowledge is important to developing an understanding of informal carers’ work: what they do, what happens to them and what it feels like [21]. This knowledge should then be used to bring into view the institutional field that the informal carers are located in, and how it influences their work [21] (Fig. 1).

The figure shows how the research process progressed from the problematic, seen from the perspective of informal carers, to practice, which allowed a view of how institutional discourses about the carework of informal carers influence both administrators’ allocation practices and the carework provided by informal carers.

### Data collection and analyses

During September and October 2014, qualitative, semi-structured interviews were conducted with 26 informal carers caring for persons with dementia. Of these, nine were the spouses of the person with dementia, 13 were their children, and four had another relationship to the person with dementia: one sister, two cousins and one friend. Three of the informal carers were male, and 23 were female. Names used in quotations are pseudonyms. Two participants were located through healthcare workers in a nursing home. The healthcare workers handed out written information provided by the author provided, and facilitated communication with informal carers who were willing to participate in the study. A conversation was held with the informal carers before the interview to ensure that they were fully informed about the research before consenting to participate. The remaining 24 informal carers were recruited through two local newspapers. After contacting a journalist in each of the newspapers, the author was interviewed by one newspaper and was able to place a small ad in the other. Informal carers who read about the study made contact by telephone or e-mail, and received oral and written information before they agreed to participate. The participants lived in 12 different municipalities.

All of the interviews were conducted as face-to face interviews. Fifteen of the interviews took place in the informal carer’s home, six participants were interviewed in the author’s workplace, four chose to meet in a café or more neutral public place and one preferred to be interviewed in her workplace. The participants chose where the interview took place. Several of the interviews had to be *timed* or held outside their homes, because the informal carers saw it as difficult to give the interview with the person with dementia present. Several participants asked to give the interview when the person with dementia was at respite care. The interviews lasted between 50 min and 2 h.

All interviews were transcribed by the author. The data was first analysed using NVivo software, outlining categories of informal carers’ work based on categories of *what* the informal caregivers said they do. Specifically, the informal carers work was grouped in categories related to the work they do as informal carers, such as: caring, organising, investigating, filling in. During this work, it became clear that there is a gap between what the informal carers want to do and what they actually do. Although participants lived in different locations and had different challenges, there were many similarities in their stories about their situation as informal cares and their contact with formal care resources, especially regarding the gap between what they want to do and what they in fact do.

As noted earlier, in institutional ethnography people’s experiences should be used to bring into view the institutional field that their work is located in [21]. Analyses of the interviews therefore looked for traces of the institutional. These traces were mainly found in the data from the interviews where the questions were directed towards *how* and *why* the informal carers acted as they did. Through the knowledge that the participants shared, similarities in their experiences were identified. A common trace was linked to the allocation divisions in the municipalities where the participants lived. These allocation divisions were central in deciding what kind and amount of formal health and care services the person with dementia could access. The author therefore contacted some of the allocation divisions in order to follow these traces. Seven administrators working on the allocation of health and care services in five municipalities were interviewed. They were asked about their work allocating health and care services to persons with dementia and their informal carers. The municipalities were selected to ensure variation in size and number of inhabitants. The interviews with the administrators were all conducted as face-to-face interviews in the allocation division where they worked. The interviews lasted approximately 1h, and were conducted in the autumn of 2014 and spring 2015.

The interviews with the administrators were analysed in two steps. They were first categorised by what interviewees said about what they actually do. This was divided into three tasks: mapping needs for help, granting help and evaluating decisions. The analysis then looked at what interviewees said about *how* and *why* they acted as they did. This second step indicated that there are some rules or guidelines for the allocation of health and care services that are not written down or explicitly articulated within the municipalities’ policies. Nevertheless, these rules or guidelines were known by all the administrators, and there were also traces of them in the interviews with the informal carers. For example, several informal carers said that they had to do the shopping for the person with dementia. This was not necessarily because they wanted to take care of this task, but because they understood the municipality to be unable to offer this service. When the administrators were asked about this, they explained that they could arrange something if the informal carers were away or the person with dementia did not have any informal carers nearby, but usually the informal cares did it. In other words, they did offer grocery shopping or other similar practical tasks as a service, but only in exceptional cases.

That these rules or guidelines were accepted by both administrators and informal carers as “the way it is” was identified as a topic of investigation. To identify the origins of these rules and guidelines and how they are maintained, central documents regarding the allocation of health and care services and the relationship between formal and informal carers were examined in further detail.

### Tracing the discourses

Institutional ethnography urges us to look beyond the everyday world. The goal is to discover how the experiences people have in their everyday life are influenced by institutional discourses. As Smith [22] says: “It is not meant as a way of discovering the everyday world as such, but of looking out beyond the everyday to discover how it came to happen as it does.”

The term “discourse” is used in many senses, in both linguistics and sociology. In institutional ethnography, the term is based on Michael Foucault’s use of the it, as Foucault used “discourse” to describe and identify conventionally regulated practices. However, a discourse is something we participate in. In this article the term *influence* is used*,* as institutional ethnography does not see institutional discourses as a form of top-down coercive power, but as a field of relation that includes “not only texts and their intertextual conversation, but the activities of people in actual sites who produce them and use them and take up to the conceptual frames they circulate” [23]. The analytic goal in an institutional ethnography is to make visible how this happens [21].

Texts are especially interesting in the process of tracing institutional discourses, because texts are integral to how people’s local activities are influenced by institutional discourses. The constancy of the text is key here. This constancy makes texts a productive tool in tracing institutional discourses across multiple local sites [20]. This article shows that the work of informal carers and staff in the allocation divisions can be understood to be connected to institutional relations that we can find traces of in the government documents about health and care services in the municipalities [3–6, 24]. To locate the discourses, government documents about health and care services for persons with dementia and their informal carers published in the last 10 years were read. The government documents that referred to this topic were *To tell the truth about informal caregiving* [3]*, Innovation in the Care Service* [4]*, Future Care* [5]*, Forgetful, but not forgotten* [6] *and Plan for dementia care 2020* [24]*.*

As noted earlier, the interviews with the informal carers and the administrators showed that they referred to some unwritten rules or guidelines. Interestingly, these unwritten rules were much-discussed in the government documents. These central matters were categorised within the three discourses that were found to influence the work of the informal carers: Family and moral obligation, Task-specific care and Shared care. The results section of this article describes how these three discourses influence the informal carers’ work.

### Ethics

The Norwegian Data Protection Official for Research [25] was informed about the study and gave its approval (reference number 35031). All the informal carers received information about the study’s aim and scope, that participation was voluntarily and that consent could be withdrawn at every stage. Written consent was based on both oral and written information. The participants are anonymised, and the names in quotes are pseudonyms.

## Results

### Institutional discourses on the work of informal carers

**Fig. 2:**
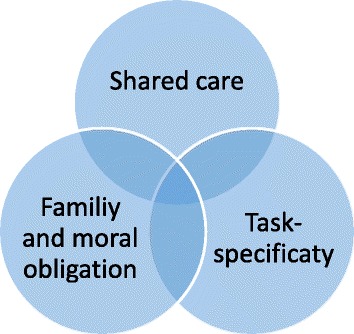
Discourses about the informal carers’ work: The figure illustrates that while these institutional discourses can be identified separately, they must also be viewed in relation to each other as the institutional field that influences the work of informal carers

Three institutional discourses on the work of informal carers have been identified: Shared care, Task- specific care, and Family and moral obligation. The figure below illustrates that while these institutional discourses can be identified separately, they must also be viewed in relation to each other as the institutional field that influences the work of informal carers (Fig. 2).

This section describes how the three discourses influence the administrators and the informal carers, and how they appear in the government documents. *Caring as a family and moral obligation* refers to a normative responsibility to care for the people we are closest to when illness strikes. Closely related to the field of moral obligation, the discourse of *shared care* points to a fundamental sharing of the responsibility for care that has traditionally been accepted as a task shared between families and the state. As an extension of the acceptance of a shared care model, certain tasks have been established as respectively the family’s and the state’s responsibility, which is here referred to as the *task-specificity discourse*.

### Caring as a family and moral obligation

In the interviews, all the informal carers expressed a great deal of care for their next of kin with dementia. They wanted to help, and they felt a responsibility to help. For some of them, it was an expected phase after many years of marriage. One of the carers said this about caring for his wife after being married for over 50 years:

*I have a responsibility to her, to make sure she is fine. I fully take on that responsibility, to make sure that her life is decent. (Ruben, informal carer)*

Consequently, some of the informal carers felt that not being able to fully care for their next of kin with dementia was a personal failure. Many feelings were involved: they felt that providing care was their responsibility, and they also worried that others would not take care of their next of kin like the carers would themselves. “It’s awful, he is to be pitied”, one of the informal carers told me. She and other informal carers I interviewed told me that they tried to postpone a major intervention by the formal system of care for as long as possible.

*I tried to spare him and look after him myself, but in the end I could not take it anymore, he was so angry, and it frightens me, it hurts so bad. (Anna, informal carer)*

Like Anna, many of the informal carers struggled with feelings of powerlessness and inadequacy. They wanted to help, but at some point they realised that they could not cope. They realised that they needed help from the public welfare services to manage everyday life.

The day the person with dementia moves to a nursing home is described as a turning point for the informal carers who have experienced it. “When they move to a nursing home, you lose some things as a carer”, one carer noted. Many carers have been tired for a long time, hoping that a place would become available soon, but at the same time the actual move is sad, and represents a major change when the day is actually there. “It’s like cutting a cord”, one informal carer said. Informal carers who are not there yet have a lot of mixed feelings. They are waiting and hoping a place will be available soon, but at the same time they dread the day it does become available because they worry about not being there for their next of kin as much as before.

Informal carers find that they first and foremost help their next of kin because they want to and feel a family and moral obligation to do so. As noted earlier, informal carers contribute to a large share of the care given to persons with dementia [5]. The government documents focus a great deal on the moral obligation informal carers feel towards the person with dementia. According to Kaasa [3], most informal carers feel a moral responsibility to help their next of kin when they get older and need care. Even though they do not have a legal obligation to contribute, this moral obligation means that it is important to ensure that the informal carers are collaborators with the formal care system. The Ministry of Health and Care Services HOD [5] and the Norwegian Directorate of Health [6] also emphasise the importance of supporting informal carers who provide care for their next of kin, offering them guidance and information about the illness and about the formal care services. In particular, the Ministry of Health and Care Services emphasises the importance of the national training programme for informal carers caring for persons with dementia as an important measure to give informal carers knowledge and support [3]. The training programme is now implemented in 80% of Norwegian municipalities, and is planned implemented in all Norwegian municipalities by the end of 2020, according to the Norwegian Dementia Plan for the period 2015–2020 [24]. The training programme is therefore a highly prioritised measure, which is also reflected in the fact that many of the informal carers in this study participated in it. However, having a training programme like this also contributes to some specific expectations about what it means to be an informal carer.

Thirteen of the 26 informal carers interviewed had participated in the training programme for informal carers who provide care for persons with dementia. The informal carers who had participated in this programme all learned something about options for health and care services. Some of them used this new knowledge to consider applying or actually apply for services they did not know existed before participating in the programme. All the informal carers who had taken the training programme signed up themselves after reading about it in the paper or hearing about it from acquaintances. However, the support and information provided in the programme were directed towards how the informal carers could contribute on terms set by the existing formal care services. Relying on the informal carers’ family and moral obligations, the training programme provides selective knowledge and presumes a particular way of providing informal care. According to the government document *To tell the truth about informal caregiving* [3], the programme seeks to give the informal carers knowledge and support, and to enable them to contribute. In this way, the training programme supports the family and moral obligation the informal carers feel, but at the same time it makes the informal carers’ contributions fit into the organisation of the formal services.

Some of the informal carers interviewed were healthcare workers or nurses themselves, or had close relatives or friends who were health professionals. This seems to have some of the same benefits as participating in the training programme for informal carers: as the quote below shows, as they were aware of a lot of the healthcare options.

*Because I used to work in healthcare myself, I knew everything I could get help with: health services, aids and so on. I think it’s much worse for those who don’t have that kind of knowledge. (Eva, informal carer)*

Like the informal carers who found support and information through the training programme, the informal carers who were healthcare workers themselves, or had close relatives or friends who were healthcare workers, also felt more able to access support that enabled them to perform informal care. Informal carers without this kind of knowledge struggled more, and took longer to find out what they could expect from the public healthcare system. The ability to perform informal care when support is also needed from public services can therefore be said to depend on having the skills to adapt to a division of labour that has already been set. This leads us to the related discourse of shared care.

### Shared care – the irreplaceable informal carers

Interviews with administrators in the allocation divisions left no doubt that the role of the informal carers is seen as important and necessary.

*I don’t know how to say this, but we can never replace the informal carers. We really can’t. And it is important to tell them that, that we can’t help with all needs, it is only the [things that are] most needed we can offer help with (Administrator 5, municipality A)*

As the quote illustrates, administrators clearly express that the contributions made by informal carers are needed and that they rely on these contributions. “There’s a gap between the expectations about public services and what we can offer” (Administrator 3, municipality L), one of them explained. He further explained that expectations are growing and have reached a level where the formal health services can never keep up. According to this administrator, this gap consists of a discrepancy between an increased standard of living and the municipalities’ mandate to offer services to safeguard the inhabitants’ basic need for health and care The administrators’ expectations about the municipality’s limited contribution is the same identified in the government document *Innovative care services* [4]. In this document the government states that finds it:

*“necessary to confront the tendency in recent years to turn health and care services into something people are to only relate to as customers and consumers (…) A one-sided consumer focus contributes to stimulating dissatisfaction and undermining confidence in public systems, when it turns out that the public services cannot deliver in line with ever-increasing and to some extent unrealistic expectations about quality and scope”*

Like the white paper, the administrators stress that the informal carers’ expectations are unrealistic and too high. “The informal carers’ expectations are too high”, several administrators noted. “They have to lower their expectations, and realise that they cannot expect the formal care services to contribute as much as they do.” The administrators do not only talk about the limitations of formal care services, they also enact these limitations. In their practice, these enactments appear as different forms of work to keep the expectations of users and informal carers on a level that makes clear that the health and care services are limited. The administrators explained that they have limited options and that they always have to try to reduce costs by not offering more than what is strictly needed. They do this by allocating services in the manner required by law, but also by ensuring that they convey the “right” expectations about the level of service the municipality can provide. “We try to be restrictive, you know, so that they understand that they can’t ask for ‘just anything’” (Administrator 2, municipality L), one of the administrators emphasised. Another administrator told me that at the time of the interview, they actually had some available places in the nursing home, but though they had people asking for places, they would not allocate them just yet:

*We try to keep the request for granting an application at the highest level possible (…) If we don’t, we risk lowering the requirements for getting a place, and then we end up with a shortage of places. (Administrator 4, municipality D)*

Several of the informal carers questioned how it is possible to manage without informal carers. “I don’t know how they make it, those who don’t have informal carers”, one said. As the administrators talked about the informal carers, it was clear that the expectation was for the informal carers’ *natural role* to be to care and to let the formal services know what their relative’s needs were. In return, the allocation division offered support, such as respite care.

*We try to listen to informal carers, especially when they tell us that they are tired, so we can offer a stay at respite care to give them a break. (Administrator 1, municipality F)*

As this quote illustrates, the allocation divisions confirmed that they saw themselves as backup for the informal carers; in other words, they limit the role of the municipality by emphasising the importance of informal carers and their contribution. Only when informal carers are prevented from making their usual contribution do the formal services see it as their task to fulfil the need for care. A quote from one of the administrators illustrates this:

*If the person with dementia does not have an informal carer at home or nearby, we monitor them more closely. In those cases, the home healthcare nurses pay closer attention to what they may need help with. (Administrator 1, municipality F)*

However, several of the informal carers find the formal care services’ practice of placing themselves second in line to be tiring, as the informal carers are entrusted with an overwhelming responsibility for their next of kin. The practice may also imply that the informal carer is the one in need. One of the informal carers put it like this:

*Respite care, as they call it… by this offer, they indicate that I’m the one who can’t manage (…) that they are kind enough to help me manage my responsibilities. (Berit, informal carer)*

As the quote shows, the practice of respite care can be seen as a provocation. Berit, the informal carer in the quote above, contributes a large part of her next of kin’s care. The way the municipality produces her as a needy service user provokes her. In a way, the shared care becomes formalised without the consent of the informal carers.

### Organising care within a framework set by the public services: a task-specificity model

The informal carers’ stories are quite similar: the illness gets worse and the person with dementia needs more help. The need for help includes more and more tasks and becomes more comprehensive. The informal carers interviewed for this research project said that as the person with dementia’s needs expanded, the carer got in touch with the allocation division in order to get help. Yet the informal carers’ stories are characterised by a sense that they are “filling in” and helping with the tasks they cannot get someone else to help with.

*Sara (informal carer): I did the grocery shoppingInterviewer: Did you choose to do it?Sara: No, there were no other options. The home healthcare service won’t do it, they don’t want to do anything with money. So I had to help them with everything that involved money.*

Many of the informal carers find this to be tiring. They want to help, but some of them also say that they simultaneously feel tied up. They find that there are no collaborative practices or overall plan for the help the person with dementia needs, which results in an unpredictable daily life for the informal carer. The informal carers are fitting the pieces of the puzzle, and where a piece is missing, they fill it themselves.

The administrators in the allocation divisions also talked about tasks that the informal carers usually do. This included practical tasks, such as grocery shopping, shopping for clothes, and transportation to or from respite care, medical appointments, and dentists’ appointments. The informal carers said that most of these tasks have in common that they are not on the allocation division’s list of available services.

The administrators said that they rarely found that informal carers performed these tasks inadequately, and consequently it had become part of an apparently natural division of tasks:

*Transportation issues are not something we hear a lot about. It is not a service we offer, our job is to allocate the respite care or short term stay. Usually the informal carers drive them there, I think. But I assume that the nursing home or the primary doctor arranges transportation if they need an ambulance or help getting there otherwise. (Administrator 4, allocation division D)*

An administrator in another allocation division said that in their municipality, the kind of service granted determines whether transportation is “included”. If the person with dementia is granted a short term stay in a nursing home, transportation is not included. However, if the short term stay in the same nursing home is granted as *respite care*, transportation is arranged by the municipality. Otherwise, the informal carer usually transports the patient. This expectation of a task-specificity model is also traceable in the government document *To tell the truth about informal caregiving*, in which [3] says that it looks like the family and the public welfare services have agreed on this division of work:

*“It looks like the family and the public services have agreed to a division of labour for the care of the very old, where the public services generally take care of the most burdensome care responsibilities, and the family provides practical help and supervision, and in emergency situations they also help the elderly so that they receive help from the public services when needed.”*

However, most of the informal carers did not see this task model as a good agreement. Several of the informal carers also felt used by the municipality. “It eats you up. You want it to be done right, but no one else seems to want the same”, one carer said. Another carer said that she found that the municipality took advantage of exhausted informal carers:

*You know, people can’t take it anymore, they are exhausted. And the municipality takes advantage of it, because in the end people give up and stop nagging. (Louise, informal carer)*

At this stage, the informal carers felt alone and at a loss. Several of the informal carers said they had to reduce their hours at work, either through early retirement or by taking sick leaves. This was not necessarily because they wanted to do so, but they could not see other option.

## Discussion

**Fig. 3:**
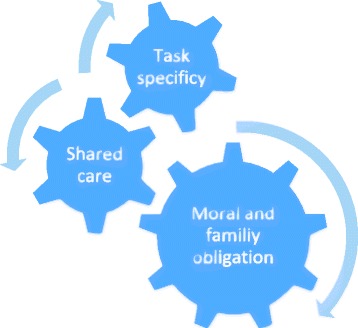
The interdependence of the three discourses: The “big wheel” in this combination of the three discourses is the moral and family obligation. The discourse of informal carers’ moral and family obligation contributes to maintaining the shared care and task specificity model

As noted in the introduction to this article, there are no legal obligations that require the informal carers to care for their next of kin. At the same time, the informal carers’ efforts are essential, and government documents refer to the informal carers as collaborators [4]. The informal carers interviewed in this study all contributed a large part of the care for their next of kin. However, the findings show that the informal carers found that the formal care system set the limitations and the framework for both the care their next of kin with dementia received and for the informal carework. The results section described how three institutional discourses of informal carers’ work (caring as a family and moral obligation, shared care and the task-specificity model) frame the informal carers’ work. This discussion section explores how these institutional discourses work together in the institutional field they operate in. In line with institutional ethnography, the aim is to see how “people’s actions are coordinated and concerned by something beyond their own motivations and intensions” [23] (Fig. 3).

The “big wheel” in this combination of the three discourses is the moral and family obligation. The discourse of family and moral obligation has a long history, and is originally based on the interpersonal relationship between family members. Unlike the discourse of shared care and task specificity, which can be said to have been created within the organisation of the modern welfare state, the discourse of family and moral obligation has roots in to the foundational values of Norwegian society. However, the discourse of family and moral obligation reflects an important relationship that organises and shapes the basis for informal carers work, including in relation to the public welfare services. The discourse is active through the practices it engages in, and it contributes to maintaining the shared care and task-specificity model. This happens through the allocation divisions’ practices that are shaped by these discourses, but also through the informal carers’ actions as they withhold the amount of care they contribute, in spite of the public services’ expectations.

The allocation divisions base their allocation of services on the informal carers’ moral and family obligation being performed in a specific way. To be an informal carer entails not just being a next of kin, but also being expected to perform care in a specific way. The training programme for informal carers shows quite clearly what these expectations are. The informal carers get particular forms of knowledge and skills through participating in these classes, and, not surprisingly, these are skills and knowledge that are consistent with the discourse of shared care and task specificity. Most of the informal carers participating in this study had taken courses for informal carers caring for persons with dementia, or were healthcare workers themselves. It is reasonable to assume that as these informal carers know more about the public welfare system, they also know more about their rights and options. Their process of accessing formal services is therefore not as heavily characterised by searches for options and repeated rejections. These informal carers did not seem to receive more extensive services, but the process of finding and accessing formal services seemed to be easier and involved fewer rejections.

Yet most of the informal carers found that their efforts were expected, and in some cases that the expectations were too high. They felt used by the municipality, and they were tired of repeating requests for help. To understand the informal carers’ frustration, these expectations by the municipality must be viewed in light of the informal carers’ sense of having a moral obligation to care for their next of kin. When the informal carers find that their applications for help are answered with inadequate and insufficient action, this leads to inadequate care for the person with dementia. It is also insulting to the informal carers’ views of adequate, respectful care and the moral obligation they feel to fulfil this. When the road towards care for their next of kin seems insuperable, the feeling of helplessness and inadequacy grows.

According to Smith [20], institutional discourses are not to be understood as predictive powers, but as “providing the terms under which what people do becomes institutionally accountable.” To understand how the three discourses of informal carers’ work frame the informal carers’ actions, it might therefore be useful to take a closer look at the relationship between informal carers and the allocation divisions. This relationship can be seen as a clientification process, where the individual’s situation and needs are expressed and understood within the framework of the formal welfare system [26]. As the results show, the allocation divisions’ practices were characterised by what can be seen as a supportive strategy, placing the divisions as secondary to the informal carers. The administrators explained their reluctance to allocate services by the necessity of preventing high costs, and as a way to encourage the elderly to live longer at home. In other words, they emphasised the importance of informal carers’ contribution to shared care. Both arguments are found in the government documents on care for the elderly in Norway [3–6]. However, most of the informal carers found this supportive strategy to be quite demanding. As demonstrated in this article, the informal carers found that some tasks were expected of them, such as grocery shopping and things involving money. It was not a written rule, but many of the informal carers had the same experience, despite data having been collected from 12 different municipalities. For instance, there appears to be a tradition of informal carers performing practical tasks: both participants in this study and government documents describe this; Kröger [2] calls this approach a *task-specificity model.* This does not appear to merely be a tradition. The informal carers found that the tradition of helping elderly family members had been transformed into an expectation placed on the informal carers. The results also show that this expectation that practical tasks will naturally be performed by informal carers forms part of the allocation divisions’ practices. This institutional discourse about informal carers’ contribution to shared care impacts informal carers through the practices of the allocation division. For informal carers, the problems seem to emerge when the formal services act upon this “agreement” as if it is a formal agreement, while the informal carer does not. Informal carers find the *task-specificity model* to be problematic, as it has become not only a product of the informal carers’ moral obligation, but also a part of the expectations from the welfare state’s formal care system.

Yet the informal carers still fulfil their expected role in the shared care, including the specific tasks necessary to meet the person with dementia’s need for care. The allocation division seems to understand this to be a result of their restrictive practice, which focuses on support for informal carers. In contrast, this article argues that the informal carers’ experiences of the demands that are placed on them instead need to be understood in light of the influence of care as a family and moral obligation. The results show that informal carers do feel that they have *a family or moral obligation* to care for their next of kin with dementia. This is not a surprising finding, as is described in the introduction to this article. Nevertheless, it is important to note that the informal carers feel this moral obligation *to their next of kin*, not to the formal welfare system. The moral obligation of care, as the informal cares describe it, is therefore only an obligation that exists within the relationship between the person with dementia and the informal carer. The problem for informal carers seems to arise when this moral obligation of care is transformed into expectations of an agreement between the informal carer and the formal welfare system. In this way, the institutional discourses of caring as a family and moral obligation and of shared care impacts the informal carers through the work of the allocation divisions. In other words, the institutional discourse of shared care is maintained by the informal carers acting on their sense of having a moral and family obligation, not by a restrictive allocation practice.

Sampling may represent a limitation of this study. After reading about the study in the newspaper, most of the participants contacted the researcher themselves, with the goal of participating in the study. This may be of significance for what they said and their motivation to participate may have affected what they said. Many of the participants said that they wanted to participate in the study because their personal experiences had made them aware of the importance of this field of research. The sampling method may also have contributed to recruiting participants who all wanted help from the formal care system. This may have excluded persons who are reluctant to access these services.

## Conclusions

A central point of an analysis based on institutional ethnography is to see how discourses organise peoples’ experiences, but to never lose focus on the subject at the centre of the study [23]. In this study, the subject at the centre is the informal carers, and it is therefore appropriate to return to some practical implications of the study at the end of this article. According to government documents such as *Future care* [5], informal carers provide care, including by helping out with personal and practical everyday tasks. The Ministry of Health and Care Services [5] also refers to studies that show that caring for elderly next of kin does not negatively affect the informal carers’ life. Nevertheless, these studies have in common that they are based on the practical performance of care, or what Etters, Goodall [8] refer to as caregiving. This does not include the psychological distress that might follow. The results in this study indicate that it is inappropriate to draw a line between caregiving and caregiver burden: they are both a result of the carework informal carers do. As meeting the need for care depends on informal carers contributing considerable amounts of work, all of the efforts informal carers make must be made visible and acknowledged. All of the efforts informal carers make to manage everyday life for the person with dementia and themselves are forms of care, whether it is helping their mother shower or their commitment, anger and frustration in trying to get respite care. To understand the efforts informal carers actually make, and to fully utilise this as part of society’s total care resources, a generous concept of care should be used. The carework done by informal carers cannot be seen as tasks that are separate from the carers’ role as next of kin, but must be seen as a result of the moral and family obligation they act upon and as a result of these traditional moral obligations having been turned into expectations through the influence of institutional discourses of informal carers’ work. The informal carers’ moral obligation is essential to their carework, and formal services should emphasise this obligation. However, treating this obligation as a formal agreement can work against the purpose of shared care, and may undermine the effort the informal carers actually make.

The results suggest that further research should ask questions that challenge how services are distributed and allocated, rather than focusing on improving the informal carers’ ability to navigate the system or enable them to be better informal carers in other ways. The results in this study suggest that the fears of informal carers’ unrealistic expectations are grossly exaggerated. Family care is unlikely to disappear; their family- and moral obligations stand strong. The problematic outcomes for the informal carers are not caused by their trying to avoid caring for their next of kin. The problematic outcome must rather be seen in light of a forced change in their role from family carer driven by family and moral obligations to being treated as a pawn within the framework of public services. At a time when many welfare states in the western world make necessary cuts to balance budgets, this study’s exploration of how institutional discourses influence the outcome of informal carers’ work is worth paying attention to. To fully utilise the total amount of a society’s care resources requires a careful allocation and distribution of formal care services in a way that emphasises informal carers’ contributions, rather than restrain them.
